# Involvement in a Personal Injury Claim Is Associated With More Pain and Delayed Return to Work After Elective Nonsurgical or Surgical Treatment for Hand or Wrist Disorders: A Propensity Score–matched Comparative Study

**DOI:** 10.1097/CORR.0000000000002410

**Published:** 2022-09-26

**Authors:** Lisa Hoogendam, Mark Johannes Willem van der Oest, John Sebastiaan Souer, Ruud Willem Selles, Steven Eric Ruden Hovius, Reinier Feitz

**Affiliations:** 1 Department of Plastic, Reconstructive, and Hand Surgery, Erasmus Medical Center, Rotterdam, the Netherlands; 2 Department of Rehabilitation Medicine, Erasmus Medical Center, Rotterdam, the Netherlands; 3 Xpert Clinics, Hand and Wrist Care, Zeist, the Netherlands; 4 Department of Plastic, Reconstructive, and Hand Surgery, Radboud University Medical Center, Nijmegen, the Netherlands

## Abstract

**Background:**

A small proportion of patients treated for a hand or wrist condition are also involved in a personal injury claim that may or may not be related to the reason for seeking treatment. There are already indications that patients involved in a personal injury claim have more severe symptoms preoperatively and worse surgical outcomes. However, for nonsurgical treatment, it is unknown whether involvement in a personal injury claim affects treatment outcomes. Similarly, it is unknown whether treatment invasiveness affects the association between involvement in a personal injury claim and the outcomes of nonsurgical treatment. Finally, most studies did not take preoperative differences into account.

**Questions/purposes:**

(1) Do patients with a claim have more pain during loading, less function, and longer time to return to work after nonsurgical treatment than matched patients without a personal injury claim? (2) Do patients with a personal injury claim have more pain, less function, and longer time to return to work after minor surgery than matched patients without a personal injury claim? (3) Do patients with a personal injury claim have more pain, less function, and longer time to return to work after major surgery than matched patients without a personal injury claim?

**Methods:**

We used data from a longitudinally maintained database of patients treated for hand or wrist disorders in the Netherlands between December 2012 and May 2020. During the study period, 35,749 patients for whom involvement in a personal injury claim was known were treated nonsurgically or surgically for hand or wrist disorders. All patients were invited to complete the VAS (scores range from 0 to 100) for pain and hand function before treatment and at follow-up. We excluded patients who did not complete the VAS on pain and hand function before treatment and those who received a rare treatment, which we defined as fewer than 20 occurrences in our dataset, resulting in 29,101 patients who were eligible for evaluation in this study. Employed patients (66% [19,134 of 29,101]) were also asked to complete a questionnaire regarding return to work. We distinguished among nonsurgical treatment (follow-up at 3 months), minor surgery (such as trigger finger release, with follow-up of 3 months), and major surgery (such as trapeziectomy, with follow-up at 12 months). The mean age was 53 ± 15 years, 64% (18,695 of 29,101) were women, and 2% (651 of 29,101) of all patients were involved in a personal injury claim. For each outcome and treatment type, patients with a personal injury claim were matched to similar patients without a personal injury claim using 1:2 propensity score matching to account for differences in patient characteristics and baseline pain and hand function. For nonsurgical treatment VAS analysis, there were 115 personal injury claim patients and 230 matched control patients, and for return to work analysis, there were 83 claim and 166 control patients. For minor surgery VAS analysis, there were 172 personal injury claim patients and 344 matched control patients, and for return to work analysis, there were 108 claim and 216 control patients. For major surgery VAS analysis, there were 129 personal injury claim patients and 258 matched control patients, and for return to work analysis, there were 117 claim and 234 control patients.

**Results:**

For patients treated nonsurgically, those with a claim had more pain during load at 3 months than matched patients without a personal injury claim (49 ± 30 versus 39 ± 30, adjusted mean difference 9 [95% confidence interval (CI) 2 to 15]; p = 0.008), but there was no difference in hand function (61 ± 27 versus 66 ± 28, adjusted mean difference -5 [95% CI -11 to 1]; p = 0.11). Each week, patients with a personal injury claim had a 39% lower probability of returning to work than patients without a claim (HR 0.61 [95% CI 0.45 to 0.84]; p = 0.002). For patients with an injury claim at 3 months after minor surgery, there was more pain (44 ± 30 versus 34 ± 29, adjusted mean difference 10 [95% CI 5 to 15]; p < 0.001), lower function (60 ± 28 versus 69 ± 28, adjusted mean difference -9 [95% CI -14 to -4]; p = 0.001), and 32% lower probability of returning to work each week (HR 0.68 [95% CI 0.52 to 0.89]; p = 0.005). For patients with an injury claim at 1 year after major surgery, there was more pain (36 ± 29 versus 27 ± 27, adjusted mean difference 9 [95% CI 4 to 15]; p = 0.002), worse hand function (66 ± 28 versus 76 ± 26, adjusted mean difference -9 [95% CI -15 to -4]; p = 0.001), and a 45% lower probability of returning to work each week (HR 0.55 [95% CI 0.42 to 0.73]; p < 0.001).

**Conclusion:**

Personal injury claim involvement was associated with more posttreatment pain and a longer time to return to work for patients treated for hand or wrist disorders, regardless of treatment invasiveness. Patients with a personal injury claim who underwent surgery also rated their postoperative hand function as worse than similar patients who did not have a claim. Depending on treatment invasiveness, only 42% to 55% of the personal injury claim patients experienced a clinically relevant improvement in pain. We recommend that clinicians extensively discuss the expected treatment outcomes and the low probability of a clinically relevant improvement in pain with their personal injury claim patients and that they broach the possibility of postponing treatment.

**Level of Evidence:**

Level III, therapeutic study.

## Introduction

Patients involved in a personal injury claim seem to have worse symptoms before treatment and poorer outcomes after treatment for hand or wrist conditions than patients without a personal injury claim. Personal injury claim involvement may comprise work-related injury [11], traffic accidents [30], and medical malpractice [29]. Specifically, work-related injuries have been studied frequently [11, 15]. A meta-analysis of 62 studies on the surgical treatment outcomes of patients with work-related upper extremity injuries who were involved in a personal injury claim showed that these patients reported more pain and less function after surgery than patients without such a claim [11].

Although multiple studies have shown an association between personal injury claims and worse treatment outcomes in general, there still remain questions regarding the association between involvement in a personal injury claim and the treatment outcomes of hand and wrist disorders. Because previous studies [11, 15] mainly focused on the outcomes of surgical treatment, it is currently unknown whether this association is also present for patients receiving nonsurgical treatment for hand and wrist conditions. Additionally, Fujihara et al. [11] demonstrated that a considerable number of studies assessing personal injury involvement only reported postoperative treatment outcomes compared with a pre- and posttreatment change. It has been hypothesized that patients are more likely to start a personal injury claim when treatment outcomes are poor, thus explaining the association of personal injury claim involvement and poorer treatment outcomes [15]. Similarly, there are indications that a difference in preoperative symptom severity explains this association [4]. Therefore, it would be relevant to compare treatment outcomes of patients with personal injury claim involvement to patients that were similar before treatment.

Therefore, we asked: (1) Do patients with a claim have more pain during loading, less function, and longer time to return to work after nonsurgical treatment than matched patients without a personal injury claim? (2) Do patients with a personal injury claim have more pain, less function, and longer time to return to work after minor surgery than matched patients without a personal injury claim? (3) Do patients with a personal injury claim have more pain, less function, and longer time to return to work after major surgery than matched patients without a personal injury claim?

## Patients and Methods

In this matched comparative study using data from a longitudinally maintained database, we investigated whether involvement in a personal injury claim is associated with pain, hand function, and time to return to work after nonsurgical treatment, minor surgery, or major surgical treatment for hand or wrist disorders.

### Setting

Data were collected from December 2012 to May 2020 at Xpert Clinics. Xpert Clinics has more than 25 locations and employs 23 European board-certified hand surgeons and more than 100 hand therapists.

As part of routine outcome measurements, all patients were invited to complete the VAS for pain and hand function before treatment and both the VAS and a questionnaire about return to work after treatment. This cohort and data collection have recently been described in more detail [31].

In the Netherlands, a personal injury claim procedure generally takes 1 to 3 years. Patients involved in a personal injury claim can be compensated for the loss of (future) income, (future) medical costs, and general compensatory damages. Most medical costs are reimbursed through basic health insurance, which covers general practitioner visits, medical specialty care, hospital stay, most medicine, and a limited number of occupational therapy sessions. With the exception of general practitioner visits, there is a compulsory deductible of USD 392 before healthcare costs are reimbursed by healthcare insurance. Physical therapy, including hand therapy, is not reimbursed under basic healthcare insurance.

### Participants

We included patients whose involvement in a personal injury claim was known and who completed the VAS questionnaire on pain and hand function before treatment. We then divided patients into three subcohorts: patients who were treated nonsurgically (follow-up at 3 months); patients receiving minor surgery, such as trigger finger release (follow-up at 3 months); and patients receiving major surgery, such as triangular fibrocartilage complex (TFCC) reinsertion (follow-up at 12 months) (Supplementary Table 1; http://links.lww.com/CORR/A943). We excluded patients if they did not provide data for at least one of the outcome measures (VAS or return to work) at the last measurement. Additionally, we excluded patients who received a rare treatment (which we defined as fewer than 20 observations in our cohort).

**Fig. 1:**
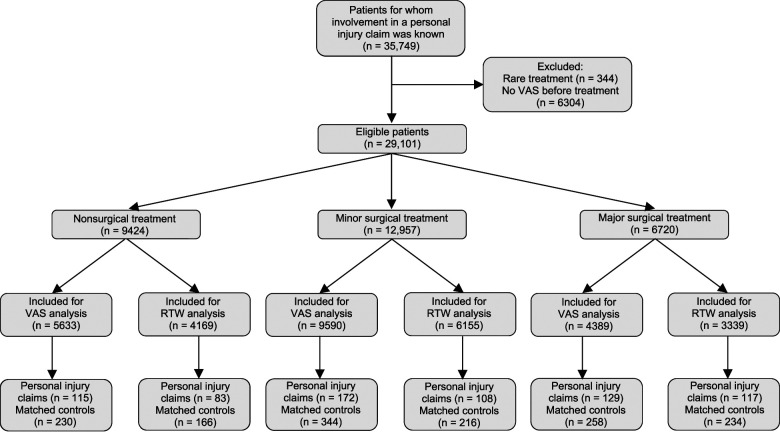
This flowchart shows the patients who were included in this study. The inclusion criteria differed for the VAS analyses (patients who completed the VAS prior to treatment and at final follow-up) and the return-to-work (RTW) analyses (employed patients who completed VAS prior to treatment), leading to some overlap in patients for these analyses. Therefore, the number of patients per treatment type can be lower than the sum of patients included in the VAS analyses and RTW analyses.

During the study period, 29,101 patients were eligible for participation in this study (Fig. 1). The mean age was 53 ± 15 years, and 64% (18,695 of 29,101) were women. Forty-five percent (12,957 of 29,101) of patients underwent minor surgical treatment, and 2% (651 of 29,101) of patients were involved in a personal injury claim. We found differences in patient characteristics and VAS scores before treatment between patients with a personal injury claim and those without one (Table 1). Patients involved in a personal injury claim were typically younger, had a shorter duration of symptoms, and performed moderate or heavy physical labor more often than those who were not involved in a claim. Moreover, patients with a personal injury claim received major surgical treatment more often and scored worse regarding pain and hand function before treatment.

**Table 1.** Patient characteristics and VAS scores before treatment for patients with and without involvement in a personal injury claim

Parameter	Patients with a personal injury claim (n = 651)	Patients without a personal injury claim (n = 28,450)	p value
Age in years	46 ± 15	53 ± 15	< 0.001
Women	62 (406)	64 (18,289)	0.57
Duration of symptoms in months	17 ± 31	21 ± 46	0.02
Type of work			0.003
Unemployed	28 (185)	34 (9782)	
Light physical labor	28 (183)	28 (8021)	
Moderate physical labor	28 (185)	26 (7286)	
Heavy physical labor	15 (98)	12 (3361)	
Dominant side			0.44
Left	10 (64)	9 (2477)	
Right	86 (563)	88 (25,070)	
Ambidextrous	4 (24)	3 (903)	
Type of treatment			< 0.001
Nonsurgical treatment	29 (189)	32 (9235)	
Minor surgery (such as, release of the first extensor compartment)^a^	40 (259)	45 (12,698)	
Major surgery (such as, TFCC reinsertion)^a^	31 (203)	23 (6517)	
VAS pain during load	66 ± 24	56 ± 28	< 0.001
VAS hand function	43 ± 24	51 ± 26	< 0.001

Data presented as mean ± SD or % (n).

a We also categorized all treatments as minor or major surgery (Supplementary Table 1; http://links.lww.com/CORR/A943).

Two percent (115 of 5633) of the nonsurgically treated patients who completed the VAS before and after treatment were involved in a personal injury claim and were matched to 230 patients without a personal injury claim for VAS analyses. After matching, there was a difference (defined as an SMD ≥ 0.10) in gender, dominant side, and treatment between the matched patients with a claim and those without (Supplementary Fig. 1; http://links.lww.com/CORR/A944), indicating that additional adjustment was required. Additionally, 2% (83 of 4169) of the nonsurgically treated patients who completed the VAS questionnaire before treatment and the questionnaire about return to work and who were employed were involved in a personal injury claim; we matched them to 166 patients without a claim for return-to-work analyses. After matching, there was a difference in treatment between matched patients with and without a claim (Supplementary Fig. 2; http://links.lww.com/CORR/A945).

For the minor surgery subcohort, 2% (172 of 9590) of patients were involved in a personal injury claim and were matched to 344 patients without a claim for VAS analyses. After matching, there was a difference in gender, dominant side, and treatment (Supplementary Fig. 3; http://links.lww.com/CORR/A946). For the return-to-work analyses, 2% (108 of 6155) of patients were involved in a claim and were matched to 216 patients without a claim. After matching, there was a difference in the type of occupation and treatment (Supplementary Fig. 4; http://links.lww.com/CORR/A947).

For the major surgery subcohort, 3% (129 of 4389) of patients were involved in a personal injury claim and were matched to 258 patients without involvement in a claim for the VAS analyses. After matching, there was a difference in treatment (Supplementary Fig. 5; http://links.lww.com/CORR/A948). For the return-to-work analyses, 4% (117 of 3339) of patients were involved in a claim and were matched to 234 patients without a claim. After matching, there was a difference in symptom duration, VAS function, and treatment (Supplementary Fig. 6; http://links.lww.com/CORR/A949).

### Data Collection

Patients were invited to complete the VAS questionnaire before and after treatment [31]. Patients were asked to score five outcome domains (average pain, pain during load, pain at rest, hand function, and satisfaction with the hand) on a VAS (scores range from 0 to 100). For pain, 0 indicates no pain and 100 indicates extreme pain; for function and satisfaction, 0 indicates poor function and 100 indicates optimal function [20]. For this study, we used the subscales of VAS pain during load and VAS hand function, which we consider the most relevant for patients with hand or wrist disorders. After treatment (at 3 months for nonsurgical treatment and minor surgery or at 12 months for major surgery), patients were invited to complete the VAS questionnaire again. The minimum important change (MIC) of the VAS pain during loading was reported as 13 for nonsurgical treatment and 23 for surgical treatment. For hand function, it was reported as 10 for nonsurgical treatment and 19 for surgical treatment [19].

In addition, patients who were employed before treatment were asked to complete a questionnaire on return to work after treatment [34]. Return to work was defined as working at least 50% of a patient’s contractual hours and performing normal working activities.

### Sample Size

The study sample was determined by the number of patients treated during the study period. For each subcohort and outcome, we calculated effect sizes to assess whether we had a sufficient number of patients for each analysis to detect a medium-sized effect (Cohen d 0.50) with 80% power [6]. The significance level was set at 0.017 to account for multiple testing of three outcomes for each subcohort. With the available number of patients, we were able to detect effect sizes ranging from 0.35 to 0.37, indicating that we had sufficient power to detect a medium-sized effect in the analyses.

### Matching

We matched patients based on patient characteristics (age, gender, occupation, and dominant side), baseline VAS scores, and treatment to compare patient-reported outcome measure scores at the last measurement for patients with and without personal injury claims. We used 2:1 propensity score matching to compare treatment outcomes, where we matched two patients without personal injury claims to one patient with a personal injury claim [3]. The number of patients differed for each analysis because not all patients completed all questionnaires. Therefore, we performed matching for each analysis separately. After matching, we calculated the standardized mean difference (SMD) between matched patients with and without personal injury claims to assess whether the propensity score matching was successful. The SMD should be below 0.10 for all variables for matching to be considered successful [25].

### Ethical Approval

Institutional review board approval was obtained from the ethics committee of the Erasmus Medical center (MEC-2018-1088). All patients provided written informed consent.

### Statistical Analyses

We used t-tests to compare normally distributed continuous variables. For nonnormally distributed variables, we used Wilcoxon tests. We compared categorical outcomes using the chi-square test.

When the SMD was greater than 0.10 for one or more variables after matching, linear regression (for VAS scores) or a Cox proportional hazards model (for return to work) was applied [25].

All analyses were performed in R, version 3.6.3. For all analyses, p < 0.017 was considered statistically significant because of multiple testing corrections. The R code used for the analyses is available online at https://doi.org/10.5281/zenodo.5607378 [17].

## Results

### Nonsurgical Treatment

**Fig. 2:**
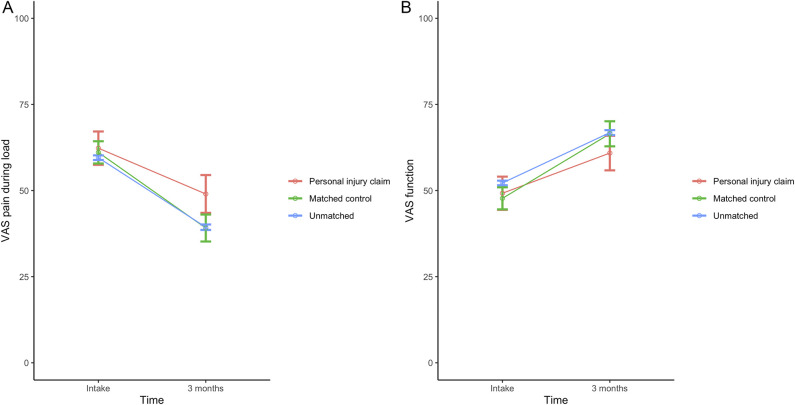
**A-B** These graphs show the mean VAS scores for (**A**) pain and (**B**) hand function during load over time after nonsurgical treatment. The error bars represent the 95% CI. Values are shown for patients involved in a personal injury claim, matched control patients, and unmatched patients not involved in a personal injury claim. A color image accompanies the online version of this article.

**Fig. 3:**
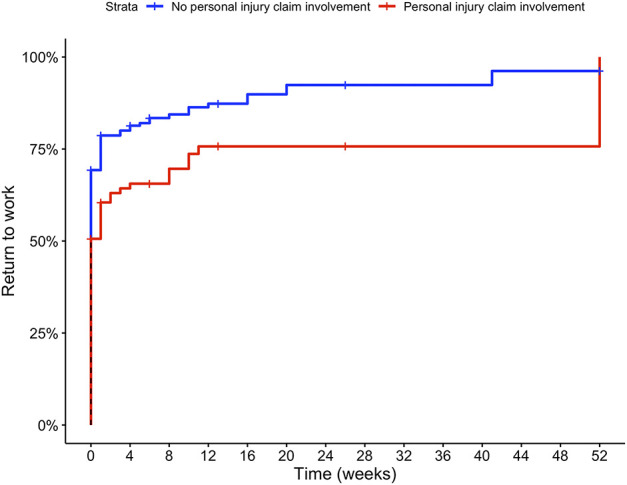
This Kaplan-Meier curve represents the time to return to work after nonsurgical treatment for patients involved in a personal injury claim and matched controls. A color image accompanies the online version of this article.

After propensity score matching and adjustment for gender, hand dominance, and treatment, at 3 months after treatment, patients with personal injury claims had more pain with loading than those without such a claim (49 ± 30 versus 39 ± 30, adjusted mean difference 9 [95% confidence interval (CI) 2 to 15]; p = 0.008) (Fig. 2A). However, there was no difference in hand function (61 ± 27 versus 66 ± 28, adjusted mean difference -5 [95% CI -11 to 1]; p = 0.11) (Fig. 2B). Forty-five percent (52 of 115) and 50% (57 of 115) of patients with personal injury claims reached the MIC of VAS pain and function, respectively (Table 2). Among employed patients, after propensity score matching and adjusting for treatment, we found that patients with personal injury claims had a 39% lower probability of returning to work each week than those without a claim (HR 0.61 [95% CI 0.45 to 0.84]; p = 0.002) (Fig. 3).

**Table 2.** Improvement over time (between baseline and 3 months) and the proportion of patients reaching the MIC for VAS pain and function [19] after nonsurgical treatment

Nonsurgical treatment	Patients with a personal injury claim (n = 115)	Matched controls (n = 230)	p value
Change in VAS pain	13 ± 34	22 ± 30	0.01
Change in VAS function	12 ± 31	19 ± 31	0.048
Reaching the MIC of VAS pain	45 (52)	55 (127)	0.10
Reaching the MIC of VAS function	50 (57)	58 (134)	0.16

Data presented as % (n); MIC = minimum important change.

### Minor Surgery

**Fig. 4:**
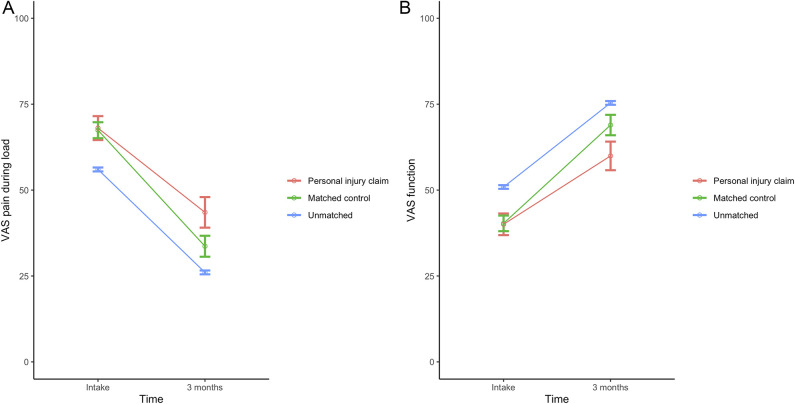
**A-B** These graphs show the mean VAS scores for (**A**) pain and (**B**) hand function during load over time after minor surgery. The error bars represent the 95% CI. Values are shown for patients involved in a personal injury claim, matched control patients, and unmatched patients not involved in a personal injury claim. A color image accompanies the online version of this article.

**Fig. 5:**
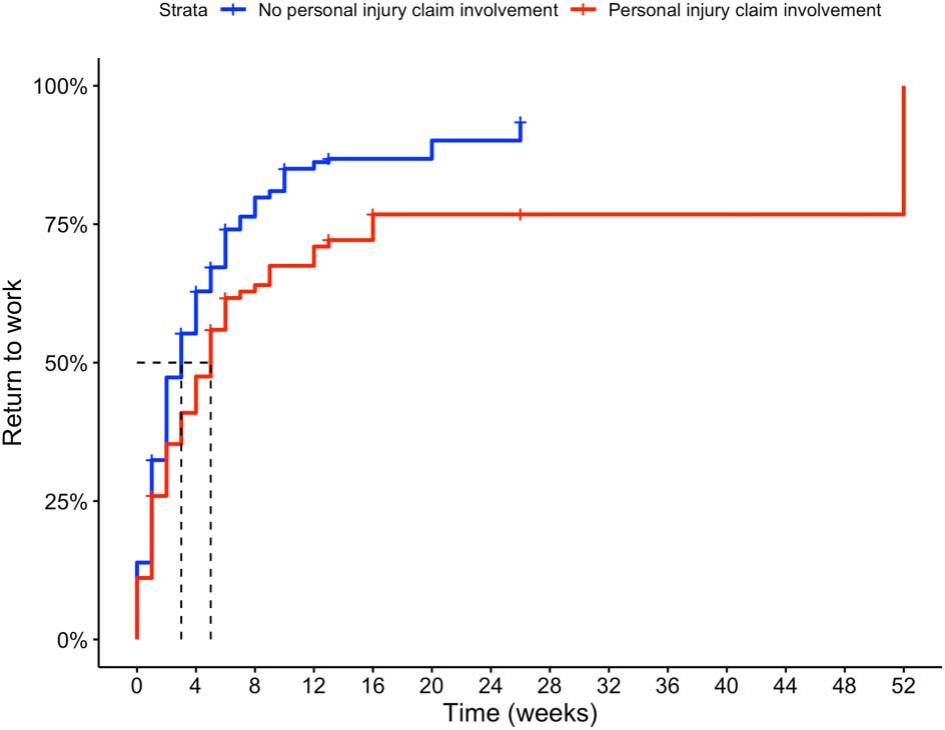
This Kaplan-Meier curve represents the time to return to work after minor surgery for patients involved in a personal injury claim and matched controls. The dashed line indicates the median time to return to work. A color image accompanies the online version of this article.

After propensity score matching and adjustment for gender, hand dominance, and treatment, at 3 months after treatment, patients with personal injury claims had more pain with loading than those without such a claim (44 ± 30 versus 34 ± 29, adjusted mean difference 10 [95% CI 5 to 15]; p < 0.001) (Fig. 4A). Additionally, patients with personal injury claims had a worse hand function than those without such a claim (60 ± 28 versus 69 ± 28, adjusted mean difference -9 [95% CI -14 to -4]; p = 0.001) (Fig. 4B). Forty-two percent (72 of 172) and 53% (91 of 172) of patients with personal injury claims reached the MIC of VAS pain and function, respectively (Table 3). Among employed patients, the median time to return to work was 5 weeks for patients with an injury claim and 3 weeks for matched patients without a claim (Fig. 5). After propensity score matching and adjusting for type of occupation and treatment, we found that patients with personal injury claims had a 32% lower probability of returning to work each week (HR 0.68 [95% CI 0.52 to 0.89]; p = 0.005).

**Table 3.** Improvement over time (between baseline and 3 months) and the proportion of patients reaching the MIC for VAS pain and function [19] after minor surgery

Minor surgery	Patients with personal injury claim (n = 172)	Matched controls (n = 344)	p value
Change in VAS pain	25 ± 30	34 ± 30	0.001
Change in VAS function	20 ± 35	29 ± 32	0.005
Reaching the MIC of VAS pain	42 (72)	61 (210)	< 0.001
Reaching the MIC of VAS function	53 (91)	64 (220)	0.02

Data presented as % (n); MIC = minimum important change.

### Major Surgery

**Fig. 6:**
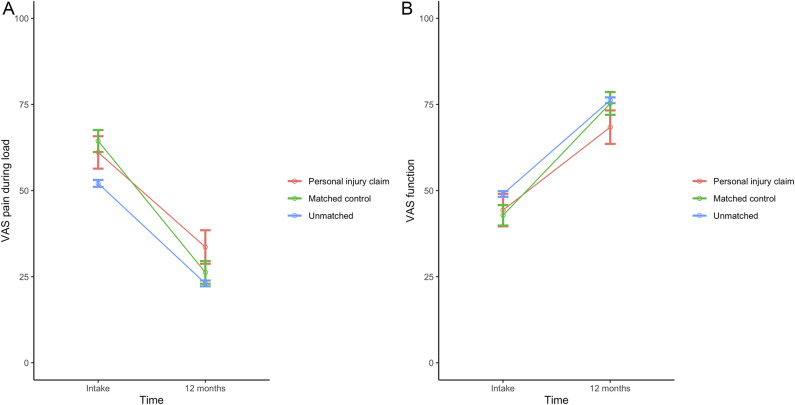
**A-B** These graphs show the mean VAS scores for (**A**) pain and (**B**) hand function during load over time after major surgery. The error bars represent the 95% CI. Values are shown for patients involved in a personal injury claim, matched control patients, and unmatched patients not involved in a personal injury claim. A color image accompanies the online version of this article.

**Fig. 7:**
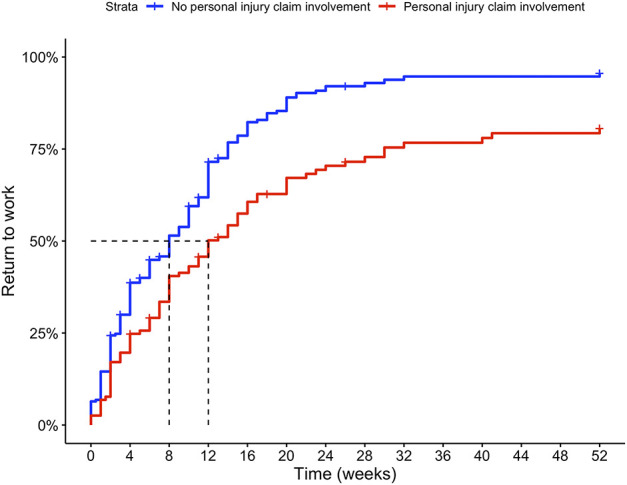
This Kaplan-Meier curve represents the time to return to work after major surgery for patients involved in a personal injury claim and matched controls. The dashed line indicates the median time to return to work. A color image accompanies the online version of this article.

After propensity score matching and adjustment for treatment, at 12 months after treatment, patients with personal injury claims had more pain with loading than those without such a claim (36 ± 29 versus 27 ± 27, adjusted mean difference 9 [95% CI 4 to 15]; p = 0.002) (Fig. 6A). Additionally, patients with personal injury claims had a worse hand function than those without a claim (66 ± 28 versus 76 ± 26, adjusted mean difference -9 [95% CI -15 to -4]; p = 0.001) (Fig. 6B). Fifty-five percent (71 of 129) and 59% (76 of 129) of personal injury claim patients reached the MIC of VAS pain and function, respectively (Table 4). Among employed patients, the median time to return to work was 12 weeks for patients with a claim and 8 weeks for matched patients (Fig. 7). After propensity score matching and adjusting for symptom duration, VAS function, and treatment, we found that patients with personal injury claims had a 45% lower probability of returning to work each week than those without a claim (HR 0.55 [95% CI 0.42 to 0.73]; p < 0.001) (Fig. 7).

**Table 4.** Improvement over time (between baseline and 12 months) and the proportion of patients reaching the MIC for VAS pain and function [19] after minor surgery

Major surgery	Patients with personal injury claim (n = 129)	Matched controls (n = 258)	p value
Change in VAS pain	29 ± 34	39 ± 33	0.004
Change in VAS function	23 ± 33	34 ± 33	0.003
Reaching the MIC of VAS pain, % (n)	55 (71)	67 (174)	0.02
Reaching the MIC of VAS function, % (n)	59 (76)	70 (180)	0.04

Data presented as % (n); MIC = minimum important change.

## Discussion

Although a small proportion of patients with hand or wrist disorders is involved in a personal injury claim, there is evidence that personal injury claim involvement is associated with worse surgical treatment outcomes. However, it was unknown whether this association is also present in nonsurgical treatment. Additionally, many studies did not take preoperative differences into account, possibly explaining this association. We found a consistent negative association between personal injury claim involvement and pain and time to return to work after nonsurgical treatment, minor surgery, and major surgery when comparing personal injury claim patients to similar patients without personal injury claim. Patients with a personal injury claim who were treated surgically also reported worse hand function. Additionally, only 42% to 55% experienced a clinically relevant improvement in pain. We suggest that clinicians discuss the expected treatment outcomes extensively with their patients who have filed personal injury claims before deciding on treatment and that clinicians should introduce the idea of postponing treatment.

### Limitations

This study has some limitations. We used the VAS to measure pain during load and hand function. Although the VAS has been validated to measure pain [20], it has not been validated to measure hand function. Using validated patient-reported outcome measures such as the Patient-Rated Wrist/Hand Evaluation or the Michigan Hand outcomes Questionnaire would have been better suited. Still, previous studies using the Disabilities of the Hand, Arm, and Shoulder questionnaire and the American Shoulder and Elbow surgeons score found similar results [2, 4, 5, 8], supporting our findings.

We were unable to determine whether the reason for treatment was related to the personal injury claim. For example, there may be a difference in symptom severity, psychological characteristics, and potential treatment outcomes between a patient involved in a personal injury claim for a broken foot who now happens to seek treatment for Dupuytren contracture compared with a patient with a personal injury claim who is undergoing a TFCC reinsertion for wrist pain after an accident. Future studies are needed to determine whether unrelated elective care can be postponed when patients have an ongoing personal injury claim.

This observational comparative study did not account for psychological factors, such as depression, pain catastrophizing, or illness perceptions. Several studies have suggested that patients with a personal injury claim have a more negative psychological profile [10, 24]. For example, patients with prolonged sick leave might have more negative illness perceptions than realistically can be expected [12]. Moreover, patients involved in a workers compensation claim have been shown to be at risk for mental illness, including depression [21, 26]. A negative psychological profile has been shown to be associated with treatment outcomes in multiple hand or wrist disorders, including carpal tunnel release [32], upper extremity fractures [1], and TFCC reinsertion [33]. Therefore, we recommend further research to identify pretreatment differences in psychological profiles and how psychological profiles of patients with personal injury claims change during treatment. This could provide insights to guide possible interventions, such as additional psychological support, social worker support, or motivational interviewing to improve treatment outcomes [14].

Additionally, there are likely differences in legal systems and claim compensation between countries in terms of the amount of compensation received or required and the duration of a personal injury claim procedure [22]. These differences might influence treatment outcomes [13]. When we compare patients in this study to patients in the United States, for example, a notable difference will be the need for medical costs compensation, which will be highly relevant for US patients but less so for Dutch patients, as these costs are mostly reimbursed under basic health insurance. Still, loss of (future) income and general compensatory damages will be relevant issues to patients in both settings. Additionally, feelings of psychological distress [7] and perceived injustice [23, 28] will affect personal injury claim patients irrespective of country, suggesting that the similarities in personal injury claims between countries may outweigh the differences. This is in line with a meta-analysis of surgical treatment outcomes of patients with workers compensation or involved in litigation [15], where it was shown that the association between compensation and worse treatment outcomes was consistently present in the United States, Canada, Europe, and Australia.

### Nonsurgical Treatment

For patients treated nonsurgically, we found that patients involved in a personal injury claim experienced more pain during load and were more likely to return to work later than patients not involved in a claim. Although it has been reported that personal injury claim involvement is associated with poorer outcomes in terms of pain and hand function for surgically treated patients [11], we find a similar association for nonsurgical treatment.

### Minor Surgery

For patients receiving minor surgery (such as trigger finger release), patients with personal injury claims had more pain, worse function, and a longer time to return to work, with a median time to return to work of 5 weeks compared with 3 weeks for controls. A difference in return to work was also reported by Dunn et al. [9], who found that patients involved in a workers compensation claim are absent from work on average 5 weeks longer. We found a smaller difference of 2 weeks’ absence for patients involved in a personal injury claim than matched patients without such a claim. Possibly, work-related injuries lead to a disturbed working relationship which negatively affects the time to return to work, which may explain the 3-week difference in the return-to-work time. We recommend a qualitative study to investigate barriers and facilitators of return to work for patients involved in a personal injury claim to guide potential interventions.

### Major Surgery

For patients receiving major surgery (such as TFCC reinsertion), patients with personal injury claims had more pain, worse function, and a longer time to return to work. In the meta-analysis by Fujihara et al. [11], it was unclear whether personal injury claims were associated with time to return to work after surgery, whereas this association was present in our study. Because the treatment invasiveness (minor or major surgery) likely affects the time to return to work, studying minor and major procedures separately might have provided a clearer overview of this association.

### Other Considerations

The differences in pain and hand function we found in all treatment groups were relatively small compared with those reported in previous studies [8, 16]. For example, a 15-point difference in VAS pain was reported after rotator cuff repair for patients with and without involvement in a personal injury claim [16], whereas we found a 9-point difference in VAS pain scores during loading after major surgery. Because it has been repeatedly shown that patients involved in a personal injury claim already report more symptoms before treatment [11, 16], correcting for baseline differences may result in a smaller difference after treatment [4], possibly explaining the difference in effect magnitude. Additionally, only 42% to 55% of patients with personal injury claims experienced a clinically relevant improvement in pain after minor and major surgery, respectively. For example, when we compare this to surgery for thumb base osteoarthritis, we find that a clinically relevant improvement in pain has been reported for 71% to 84% of patients [18, 27]. This raises the question of whether patients actually benefit from treatment while they have an ongoing personal injury claim. Future studies may evaluate whether postponing treatment is an option and how this may affect outcomes. Finally, there is an increasing interest in the development of prediction models for individualized predictions of expected treatment outcomes. Considering that personal injury claim involvement is associated with worse treatment outcomes, regardless of treatment invasiveness, we strongly recommend researchers working on prediction models for individual treatment outcomes to include personal injury claim involvement as a candidate predictor for their models.

### Conclusion

This matched comparative study found that involvement in a personal injury claim was associated with worse treatment outcomes for patients treated nonsurgically or surgically for hand or wrist disorders. Regardless of treatment invasiveness (nonsurgical treatment, minor surgery, or major surgery), the time to return to work was longer and posttreatment pain was higher for personal injury claim patients. Surgical personal injury claim patients also rated their postoperative hand function as worse than similar patients. Depending on treatment invasiveness, only 42% to 55% of the personal injury claim patients experienced a clinically relevant improvement in pain. We recommend that clinicians do the following with their patients who have personal injury claims: thoroughly discuss the expected treatment outcomes and the low probability of a clinically relevant improvement in pain as well as introduce the possibility of postponing treatment.

## Group Authorship

The Hand-Wrist Study Group includes: Richard Arjen Michiel Blomme, Dirk-Johannes Jacobus Cornelis van der Avoort, Alexander Kroeze, Jeronimus Maria Smit, Jan Debeij, Erik Taco Walbeehm, Gijs Marijn van Couwelaar, Guus Maarten Vermeulen, Johannes Pieter de Schipper, Johannes Frederikes Maria Temming, Jeroen Hein van Uchelen, Herman Luitzen de Boer, Kennard Harmsen, Oliver Theodor Zöphel, Thybout Matthias Moojen, Xander Smit, Gert-Jan Halbesma, Rob van Huis, Pierre Yves Alain Adriaan Pennehouat, Karin Schoneveld, Yara Eline van Kooij, Robbert Maarten Wouters, Joris Jan Veltkamp, Alexandra Fink, Willemijn Anna de Ridder, Joris Sebastiaan Teunissen, Jaimy Emerentiana Koopman, Nina Louisa Loos, Jak Dekker, Ward Rogier Bijlsma, Marloes Hendrina Paulina ter Stege, and Harm Pieter Slijper.

## References

[1] Al Salman A Shah R Thomas JE Symptoms of depression and catastrophic thinking attenuate the relationship of pain intensity and magnitude of incapability with fracture severity. J Psychosom Res. 2022;158:110915. 35483125 10.1016/j.jpsychores.2022.110915

[2] Atanda A Jr O'Brien DF Kraeutler MJ Outcomes after distal biceps repair in patients with workers' compensation claims. J Shoulder Elbow Surg. 2013;22:299-304. 23246274 10.1016/j.jse.2012.11.011

[3] Austin PC. Statistical criteria for selecting the optimal number of untreated subjects matched to each treated subject when using many-to-one matching on the propensity score. Am J Epidemiol. 2010;172:1092-1097. 20802241 10.1093/aje/kwq224 PMC2962254

[4] Balyk R Luciak-Corea C Otto D Do outcomes differ after rotator cuff repair for patients receiving workers' compensation? Clin Orthop Relat Res. 2008;466:3025-3033. 18784971 10.1007/s11999-008-0475-1 PMC2628237

[5] Banerjee M Balke M Bouillon B Soft tissue injury of the shoulder after single non-dislocating trauma: prevalence and spectrum of intraoperative findings during shoulder arthroscopy and treatment results. Arch Orthop Trauma Surg. 2015;135:103-109. 25394541 10.1007/s00402-014-2114-5

[6] Cohen J. Statistical Power Analysis for the Behavioral Sciences. L. Erlbaum Associates; 1988.

[7] Collie A Sheehan L Lane TJ Psychological distress in workers' compensation claimants: prevalence, predictors and mental health service use. J Occup Rehabil. 2020;30:194-202. 31646415 10.1007/s10926-019-09862-1

[8] Doherty C Gan BS Grewal R. Ulnar shortening osteotomy for ulnar impaction syndrome. J Wrist Surg. 2014;3:85-90. 25032074 10.1055/s-0034-1372519 PMC4078135

[9] Dunn JC Kusnezov NA Koehler LR Outcomes following carpal tunnel release in patients receiving workers' compensation: a systematic review. Hand (N Y). 2018;13:137-142. 28387162 10.1177/1558944717701240 PMC5950969

[10] Elbers NA Hulst L Cuijpers P Do compensation processes impair mental health? A meta-analysis. Injury. 2013;44:674-683. 22244996 10.1016/j.injury.2011.11.025

[11] Fujihara Y Shauver MJ Lark ME The effect of workers' compensation on outcome measurement methods after upper extremity surgery: a systematic review and meta-analysis. Plast Reconstr Surg. 2017;139:923-933. 28350673 10.1097/PRS.0000000000003154 PMC5373484

[12] Giri P Poole J Nightingale P Perceptions of illness and their impact on sickness absence. Occup Med (Lond). 2009;59:550-555. 19704030 10.1093/occmed/kqp123

[13] Grant GM O'Donnell ML Spittal MJ Relationship between stressfulness of claiming for injury compensation and long-term recovery: a prospective cohort study. JAMA Psychiatry. 2014;71:446-453. 24522841 10.1001/jamapsychiatry.2013.4023

[14] Gross DP Park J Rayani F Motivational interviewing improves sustainable return to work in injured workers after rehabilitation: a cluster randomized controlled trial. Arch Phys Med Rehabil. 2017;98:2355-2363. 28647549 10.1016/j.apmr.2017.06.003

[15] Harris I Mulford J Solomon M Association between compensation status and outcome after surgery: a meta-analysis. JAMA. 2005;293:1644-1652. 15811984 10.1001/jama.293.13.1644

[16] Henn RF 3rd Tashjian RZ Kang L Patients with workers' compensation claims have worse outcomes after rotator cuff repair. J Bone Joint Surg Am. 2008;90:2105-2113. 18829907 10.2106/JBJS.F.00260

[17] Hoogendam L. Personal injury claim. Available at: 10.5281/zenodo.5607378. Accessed October 28, 2021.

[18] Hoogendam L Bink T de Lange J Which tendon plasty has the best outcome? A comparison of four tendon plasty techniques in a large cohort of patients with symptomatic trapeziometacarpal osteoarthritis. Plast Reconstr Surg. 2022;150:364e-374e. 10.1097/PRS.0000000000009350 35671451

[19] Hoogendam L Koopman JE Van Kooij Y The minimal important change of four common patient-reported outcome measures for 36 different hand and wrist conditions. Clin Orthop Relat Res. 2022;480:1152-1166. 34962496 10.1097/CORR.0000000000002094 PMC9263468

[20] Kahl C Cleland JA. Visual analogue scale, numeric pain rating scale and the McGill pain questionnaire: an overview of psychometric properties. Physical Therapy Reviews. 2005;10:123-128.

[21] Kim J. Depression as a psychosocial consequence of occupational injury in the US working population: findings from the medical expenditure panel survey. BMC Public Health. 2013;13:303. 23560685 10.1186/1471-2458-13-303 PMC3635882

[22] Lippel K. Preserving workers' dignity in workers' compensation systems: an international perspective. Am J Ind Med. 2012;55:519-536. 22354856 10.1002/ajim.22022

[23] Murgatroyd DF Cameron ID Harris IA. Understanding the effect of compensation on recovery from severe motor vehicle crash injuries: a qualitative study. Inj Prev. 2011;17:222-227. 21071765 10.1136/ip.2010.029546

[24] Murgatroyd DF Casey PP Cameron ID The effect of financial compensation on health outcomes following musculoskeletal injury: systematic review. PLoS One. 2015;10:e0117597. 25680118 10.1371/journal.pone.0117597 PMC4334545

[25] Nguyen TL Collins GS Spence J Double-adjustment in propensity score matching analysis: choosing a threshold for considering residual imbalance. BMC Med Res Methodol. 2017;17:78. 28454568 10.1186/s12874-017-0338-0 PMC5408373

[26] Orchard C Carnide N Mustard C Prevalence of serious mental illness and mental health service use after a workplace injury: a longitudinal study of workers' compensation claimants in Victoria, Australia. Occup Environ Med. 2020;77:185-187. 31896616 10.1136/oemed-2019-105995

[27] Ottenhoff JSE Spaans AJ Braakenburg A Joint distraction for thumb carpometacarpal osteoarthritis: 2-year follow-up results of 20 patients. J Wrist Surg. 2021;10:502-510. 34881105 10.1055/s-0041-1728806 PMC8635830

[28] Quintner J Galbraith M. The great trade-off in workers' compensation: perceptions of injustice by those experiencing persistent pain. Pain Med. 2022;23:456-465. 33822198 10.1093/pm/pnab123

[29] Schaffer AC Jena AB Seabury SA Rates and characteristics of paid malpractice claims among US physicians by specialty, 1992-2014. JAMA Intern Med. 2017;177:710-718. 28346582 10.1001/jamainternmed.2017.0311 PMC5470361

[30] Scollay CE Berecki-Gisolf J Grant GM. Trends in lawyer use in road traffic injury compensation claims. PLoS One. 2020;15:e0231025. 32251480 10.1371/journal.pone.0231025 PMC7135282

[31] Selles RW Wouters RM Poelstra R Routine health outcome measurement: development, design, and implementation of the Hand and Wrist Cohort. Plast Reconstr Surg. 2020;146:343-354. 32740587 10.1097/PRS.0000000000007008

[32] Sun PO Walbeehm ET Selles RW Patient mindset and the success of carpal tunnel release. Plast Reconstr Surg. 2021;147:66e-75e. 10.1097/PRS.0000000000007441 33370055

[33] Teunissen JS van der Oest MJW van Groeninghen DE The impact of psychosocial variables on initial presentation and surgical outcome for ulnar-sided wrist pathology: a cohort study with 1-year follow-up. BMC Musculoskelet Disord. 2022;23:109. 35105316 10.1186/s12891-022-05045-x PMC8808973

[34] van der Oest MJW Teunissen JS Poelstra R Factors affecting return to work after surgical treatment of trapeziometacarpal joint osteoarthritis. J Hand Surg Eur Vol. 2020:46:979-984. 33287620 10.1177/1753193420978631 PMC8559178

